# Training and deployment of medical doctors in Tanzania post-1990s health sector reforms: assessing the achievements

**DOI:** 10.1186/s12960-017-0202-7

**Published:** 2017-04-04

**Authors:** Nathanael Sirili, Angwara Kiwara, Frumence Gasto, Isabel Goicolea, Anna-Karin Hurtig

**Affiliations:** 1 Unit of Epidemiology and Global Health, Department of Public Health and Clinical Medicine, Umeå University, Sweden, Umeå, SE 90185 Sweden; 2 Department of Development Studies, School of Public Health and Social Sciences, Muhimbili University of Health and Allied Sciences, P.O.BOX 65454, Dar es Salaam, Tanzania

**Keywords:** Medical doctors, Health sector reforms, Training and deployment, Human resource for health model, Planning, Shortage of doctors

## Abstract

**Background:**

The shortage of a skilled health workforce is a global crisis. International efforts to combat the crisis have shown few benefits; therefore, more country-specific efforts are required. Tanzania adopted health sector reforms in the 1990s to ensure, among other things, availability of an adequate skilled health workforce. Little is documented on how the post-reform training and deployment of medical doctors (MDs) have contributed to resolving Tanzania’s shortage of doctors. The study aims to assess achievements in training and deployment of MDs in Tanzania about 20 years since the 1990s health sector reforms.

**Methods:**

We developed a human resource for health (HRH) conceptual model to study achievements in the training and deployment of MDs by using the concepts of supply and demand. We analysed secondary data to document the number of MDs trained in Tanzania and abroad, and the number of MDs recommended for the health sector from 1992 to 2011. A cross-sectional survey conducted in all regions of the country established the number of MDs available by 2011.

**Results:**

By 1992, Tanzania had 1265 MDs working in the country. From 1992 to 2010, 2622 MDs graduated both locally and abroad. This translates into 3887 MDs by 2011. Tanzania needs between 3326 and 5535 MDs. Our survey captured 1299 MDs working throughout the country. This number is less than 40% of all MDs trained in and needed for Tanzania by 2011. Maldistribution favouring big cities was evident; the eastern zone with less than 30% of the population hosts more than 50% of all MDs. No information was available on the more than 60% of MDs uncaptured by our survey.

**Conclusions:**

Two decades after the reforms, the number of MDs trained in Tanzania has increased sevenfold per year. Yet, the number and geographical distribution of MDs practicing in the country has remained the same as before the reforms. HRH planning should consider the three stages of health workforce development conceptualized under the demand and supply model. Auditing and improvement of the HRH database is highly recommended in dealing with Tanzania’s MD crisis.

## Background

### Human resources for health crisis in a global perspective

Human resources for health (HRH) is defined as all people engaged in actions the primary intent of which is to enhance health [1]. This includes clinicians and non-clinicians such as health managers, ambulance drivers, record officers and all those involved in different projects that ensure the well-being of the people. By 2006, the world suffered a shortage of 4.25 million medical doctors (MDs), midwives, nurses and support workers [2]. The United States and Canada, which have a burden of diseases of 13%, hosts 37% of the global health workforce, while Africa, which hosts 24% of the global burden of diseases, is home to 3% only of the global health workforce [2]. Out of the 57 countries with critical shortage of HRH in the world, 36 are from sub-Saharan Africa. Shortage of HRH in sub-Saharan Africa is attributed to brain drain to high-income countries, low output from training institutions, increased disease burden and rapid population growth [3, 4]. Africa needs about half of the 4.25 million health workers required globally [2]. The rural population suffers more as within these countries migration from rural to urban areas is high among the skilled workforce [5–7].

Global efforts in redressing the HRH situation have taken place in different forms. These efforts include supporting low- and middle-income countries in training of HRH by high-income countries, the sending of health workers during emergencies or disastrous situations and the introduction by the World Health Organization (WHO) in 2010 of WHO Global Code of Practice on the International Recruitment of Health Personnel [8–12]. Although global efforts have been made to solve the HRH crisis, the fruits produced so far are not enough; this therefore calls for more in-country-specific measures [13].

### Tanzania in focus

#### Human resource for health situation and monitoring

Tanzania is among the 36 countries in sub-Saharan Africa with a critical shortage of HRH [2]. The country suffers an average shortage of 56% of skilled workforce, and the situation is worse in the private sector [14]. Tanzania not only suffers a shortage but also a maldistribution of the few HRH available in the country, as for other parts of the world, rural areas suffer more [7, 15–17]. Although 75% of the population of this east African country resides in rural areas, it is served by 55% of the nurses and 31% of the doctors available [14]. The shortage of HRH in Tanzania is believed to be a result of multiple factors including low output of highly trained cadres from the training institutions post-independence, failure to absorb all trained HRH in the country in the last two decades, brain drain and the increased disease burden [4, 15, 17]. Among the contributors of international brain drain is the failure to absorb the graduating HRH [18].

The availability of accurate data on the number of health workers is crucial in planning. The first information system, the Health Management Information System (HMIS), was introduced to the health sector in 1994–1997 [19]. This system captured all information regarding the health sector and not only on HRH. The Ministry of Health had no HRH-specific information system and therefore struggled to capture HRH information from other systems operated by other ministries [19]). This system of collecting information from other ministries was less effective, and forecasting and planning of HRH demand became difficult [20]. Starting in 2006, the government made efforts to establish a robust Human Resources for Health Information System (HRHIS) [15]. This system has two major components: the information system that collects all the information from districts, regions, consultants and referral hospitals, and the Training Institution Information System (TIIS) that collects information from all of the training institutions. The information system captures information from both the public and the private sector [19].

#### Health sector reforms

In the 1990s, Tanzania adopted health sector reforms (HSRs) that were part of the major economic and social reforms started in the mid-1980s in most developing countries. HSR is a sustained process of fundamental changes in national policies and institutional arrangements led by the government. They are designed to improve the functioning and performance of the health sector and ultimately the health status of the population. The major aims of the reforms were to bring healthcare services closer to the people and make the health system responsive [21–23].

The HSRs re-introduced the decentralization administration system and the private sector into the health sector with an emphasis on public-private partnership (PPP) [24]. Decentralization in this case refers to the transfer of power from the central government to local authorities [25]. The goal of decentralization was to bring health services closer to the people. The district therefore was made as a focal point for health programme planning and implementation [26]. Under the decentralized system, the districts were given a mandate to plan and implement health programmes, hire and fire the health workforce among other things [26]. This aimed to ensure that there was an even distribution of a skilled health workforce across the country. With this new administrative structure, the demand for MDs inevitably became high. MDs were needed in the districts to lead planning and implementation of all health programmes [26].

The PPP is a collaboration between public and private sector organizations to pool resources [financial, human, technical and information] from public and private sources to achieve a commonly agreed-upon social goal [27]. Re-enactment of the private sector into health services was made possible following an amendment of the private hospital regulation act of 1977 which banned the commercialization of health services in Tanzania [28]. The major aim of the PPP was to ensure concerted efforts and contribution of the private sector in making the health system more responsive to rising healthcare demands [26, 29].

#### Training of medical doctors

After the reforms, the demand for MDs increased and the private sector was inevitably needed to support the training of adequate number of MDs. The capacity of the Muhimbili University of Health and Allied Sciences, the only public medical training institution by then, was too low to match the rising demands. Muhimbili University (by the then a Faculty of Medicine) was admitting a maximum of only 50 medical students annually for more than 20 years since the 1970s regardless of the growing population [30]. Following the open up to a private sector, accreditation was then open to private training institutions through the Ministry of Education. From the late 1990s to 2010, four accredited private institutions which had produced at least two classes of MDs were in place [4]. There also were efforts to expand and increase enrolment in Muhimbili starting in the late 1990s [30]. The number of students enrolled in this public medical institution rose from fewer than 50 students in the 1990s to more than 200 students per year by 2010 [4, 31]. Together with in-country efforts to train MDs, the government also sent students abroad for medical training [4].

#### Deployment of medical doctors

Before the HSRs in the 1990s, the deployment of MDs, as for all other public servants in the public sector, was centralized [26]. Under this centralized system, the Ministry of Health directly posted all MDs who graduated for internship and then working stations [18].

At the early years of HSRs, the deployment system was decentralized [26]. Under the decentralized system, the districts identified their needs, searched for MDs for filling the vacancies either by advertising or by head-hunting and informed the central government, which finalized their recruitment to the districts. Despite the fact that the law required the districts to have full mandate on the deployment of MDs, central government interference continued to exist; (i) the Ministry of Finance approved district budgets and set guidelines for spending even locally collected revenue; (ii) the Civil Service Department had a central role in approving employment permits to all skilled health workers and, in collaboration with the Public Service Commission, in confirming health workers’ employment and managing their promotion, and (iii) all transfers of health workers from one district to another were managed by the ministry responsible for regional administration and local government authority (now under the President’s office) [16]. This system was not only bureaucratic but also very expensive and made it difficult for poor resource districts to get MDs. Unlike the primary objective of ensuring an even distribution of MDs, the decentralized system resulted in severe uneven distribution of MDs in the country favouring urban districts [7, 15].

In 2006, the deployment system changed to a combination of a centralized and decentralized system, called a partial centralized system. In this system, the districts identified vacancies and the Ministry of Health and Social Welfare (MoHSW) filled vacancies in collaboration with the Ministry of Finance and Economic Affairs (MoFEA), the Prime Minister’s Office Regional Administration and Local Government (PMORALG) and the President’s Office Public Service Management (POPSM) [15]. The aim of this new system, as for the previous one, was to ensure an even distribution of health workers including MDs and to mitigate the challenges faced by the decentralized system [32].

#### Deployment in the private sector

The private sector in Tanzania is comprised of three main players: Private for Profit (PfP) service providers, Non-Governmental Organizations (NGOs) and Faith-Based Organizations (FBOs) [12, 33]. The private sector has shown considerable growth since the health sector reforms. By 2012, the private sector owned 129 hospitals, which was about 54% of all hospitals present in the country. However, out of the 6663 health facilities from the dispensary to hospital level present in the country, the private sector owned only 1731, which is equivalent to 26% [34].The FBO facilities are under the Christian Social Services Commission (CSSC) and the *Baraza Kuu la Waislamu Tanzania* (National Council of Muslims in Tanzania-BAKWAKA). Out of the 1731 health facilities in the private sector, 1062 (61%) were owned by the CSSC [35]. Out of these health facilities, 101 were hospitals (two referral hospitals, two specialized hospitals, 37 Designated District Hospitals and 59 Voluntary Agency Hospitals), which forms 78% of all hospitals owned by the private sector. The other health facilities owned by CSSC include 101 health centres, 697 dispensaries and 67 health training institutions [35]. Although grossly the private sector owned only 26% of all health facilities, it is clear that it has made a significant contribution in the building of hospitals (54%) which consumes more resources than the lower-level facilities. These hospitals need a significant number of MDs to provide both clinical and management services hence creating more employment opportunities to MDs and other skilled HRH.

The CSSC is well linked to the government due to long-term collaboration where in many places there is a joint venture in service provision, where the CSSC owns facilities and management while the government second employees [35, 36]. The PfP service providers are not well linked to the government. Although the Association of Private Health Facilities in Tanzania (APHFTA) coordinates with such providers, the law does not bind the PfP services providers to surrender their information to APHFTA. The deployment system in the private sector, excluding those within the CSSC, is not well coordinated in Tanzania as each facility, organization or institution has its own deployment system.

#### Linking the reforms to shortage of medical doctors

Despite the changes introduced after the HSRs, the documented number of MDs in the country has remained critically low [37]. By 2012, the HRH situation in Tanzania had worsened; the country had a total of 0.3 MDs, nurses and midwives per 1000 people [17]. The recommended critical threshold is 2.28 MDs, nurses and midwives for 1000 people [3].

Training of one MD in Tanzania is estimated to cost 27,500 USD [38]. That is, for every 100 unemployed MDs, a minimum of 2.75 million USD is lost; this is equivalent to approximately 5.8 billion Tanzanian shillings. This cost is too high if the country does not deploy the graduating MDs especially as even the public health-training institutions are chronically underfunded [8]. Regardless of the observed increase in the number of MD graduates from training institutions, the shortage and maldistribution of MDs in the country is still high. The number of MDs documented to be in the country is almost the same as the number documented in the late 1980s before the HSRs [39]. Maldistribution is still rampant, favouring urban localities [33] with 69% of all MDs in urban areas. The total population of Tanzania is estimated at 45 million with 75% residing in rural areas [40].

In this study, we aim to assess the achievements in the training and deployment of medical doctors in Tanzania for a period of about 20 years after the HSRs of the 1990s. The choice to focus on MDs is because the cost of training of MDs is high and is largely sponsored by the Tanzanian government through grants.

## Methods

### Study setting

This study was carried out in mainland Tanzania, which is subdivided into seven geographical zones for 25 regions (21 at the time of the survey). The survey included 20 out of the 21 regions (logistical issues prevented us from reaching Manyara), 10 major health NGOs, five major health-training institutions (one public and four private) and the MoHSW (Table 2). The national hospital is found in the eastern zone (Dar es Salaam), and the zonal referral hospitals are located in northern, southern and lake zones. Bugando Medical Centre (referral for lake zone) is in Mwanza City, Mbeya referral hospital (referral for southern highlands) is in Mbeya City and Kilimanjaro Christian Medical Centre (referral for northern zone) is in Moshi town. Dar es Salaam, Mbeya, Mwanza, Tanga and Arusha are the five major cities in Tanzania. The western and southern zones are considered more rural compared to the other zones. The zones and regions are summarized in Table 1.

**Table 1:** Zonal division in Tanzania

Zone	Regions
Central zone	Dodoma and Singida
Eastern zone	Coast, Dar es Salaam and Morogoro
Lake zone	Kagera, Mara, Mwanza, Shinyanga, Simiyu^a^ and Geita^a^
Northern zone	Arusha, Kilimanjaro, Manyara and Tanga
Southern zone	Lindi and Mtwara
Southern highlands	Iringa, Mbeya, Ruvuma and Njombe^a^
Western zone	Katavi^a^, Kigoma, Rukwa and Tabora

^a^These regions are new but during the survey were part of the existing 21 regions.

### Study design

We used a case study design to allow an in-depth assessment of the achievements in the training and deployment of medical doctors in Tanzania after the 1990s health sector reforms [41].

### Conceptual model for assessing achievement in the training and deployment of medical doctors in Tanzania

**Fig. 1:**
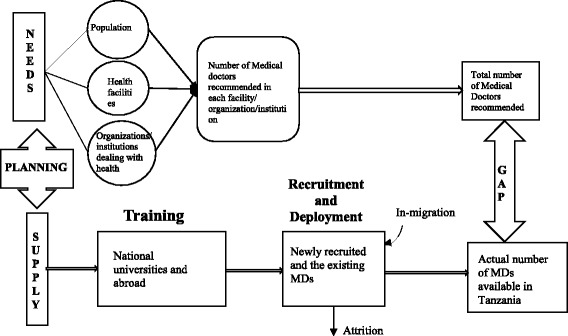
Human resource for health conceptual model for assessing the achievement in training and deployment of medical doctors in Tanzania. Source: Developed based on concepts from the health workforce development stages by WHO [42] and forecasting models for Human Resources in Health Care by Linda O’Brien Pallas [44]

We developed a human resource for health conceptual model (Fig. 1) based on concepts from the health workforce development stages proposed by the World Health Organization [42] and other relevant sources [37, 43, 44]. In this conceptual model, we considered how the Ministry of Health developed recommended staffing levels and the ideal planning of MDs training and deployment. The number of MDs recommended in Tanzania as documented in staffing levels was based on needs assessment conducted by a team of stakeholders led by the Ministry of Health and Social Welfare. The team considered the healthcare needs of the population, number and needs of health facilities available, and the needs of organizations, institutions and agencies dealing with health [45].

We conceptualized the planning of MDs by considering the needs and supply. The needs were established by considering all possible areas where MDs are recommended to work. The supply side was determined by looking at the terrains of ensuring availability of MDs using WHO’s three stages of health workforce development. The gap between the recommended and available MDs in the heath sector was obtained by taking the difference between the two.

### Data collection and data analysis

#### Number of medical doctors recommended

We conducted secondary analysis of health workers’ staffing levels to document the number of medical doctors recommended in Tanzania. We used the 2014 recommended staffing level because the previous staffing level was revised in 1999, more than 10 years before we conducted this study.

In Tanzania, the lowest facility where an MD is recommended to work is a health centre. We identified the number of MDs recommended at health centres, district hospitals, regional hospitals, regional referral hospitals and consultant hospitals. The consultant hospitals include zonal referral hospitals, specialized hospitals and the national hospital. We also identified the number of MDs required for Ministry of Health and Social Welfare agencies and allied health training institutions. We labelled the health centres as level 1 health facilities, district hospitals as level 2 health facilities, regional hospitals as level 3 health facilities and referral and consultant hospitals as level 4 health facilities.

A review of the 2012 health sector performance profile report [37] identified the number of health facilities in each level, as well as the number of allied training institutions and MoHSW agencies. The number of these facilities, training institutions and MoHSW agencies was summarized against the number of MDs required at each level in a frequency distribution table. The total number of MDs recommended in Tanzania was obtained as follows:

T_R_ = ∑^4^_*i*=1_ H_f_N_f_ + ∑ M_a_N_a_ + ∑A_i_N_i_

where

*T*_R =_ total number of MDs recommended in Tanzania

*H*_f_ = number of health facilities at each level, from level 1 to level 4

*N*_f_ = number of MDs recommended at each level of health facilities

*M*_a_ = number of MoHSW agencies

*N*_a_ = number of MDs recommended at each MoHSW agency

*A*_i_ = number of allied health training institutions

*N*_i_ = number of MDs recommended at each allied health training institution

#### Medical doctors graduated in Tanzania and abroad after health sector reforms

We reviewed graduation books from the five health training institutions available in Tanzania to document number of MDs graduated from 1992 to 2010. The involved institutions were those that had produced at least one class of MD graduates by the year 2010. These institutions were the Muhimbili University of Health and Allied Sciences, MUHAS (Public), Kilimanjaro Christian Medical University College, KCMU-College (FBO), International Medical Technology University, IMTU (PfP), Hubert Kairuki Memorial University, HKMU (PfP) and Catholic University of Health and Allied Sciences, CUHAS (FBO).

We reviewed documents from Medical Council of Tanganyika (MCT) to obtain the number of MDs trained or came from abroad and registered with MCT from 1992 to 2010 (Table 2).

**Table 2:** Data source

Training institutions	Non-governmental organizations
Catholic University of Health and Allied Sciences, Graduation books; 2008–2010	Benjamin Mkapa HIV/AIDS Foundation
Hubert Kairuki Memorial University, Graduation books; 2003–2010	Pathfinder
International Medical Technology University, Graduation books; 2001–2010 Kilimanjaro Christian Medical University College, Graduation books; 2003–2010	JHPIEGO
Muhimbili University of Health and Allied Sciences, Graduation books; 1992–2010	Strategies Insurance
Associations	AAR Insurance
Association of Private Health Facilities in Tanzania	Afya Sure
Christian Social Services Commission	Pharmaccess
MoHSW	AMREF
National Health Insurance Fund	Plan International
Tanzania Commission for AIDS	Family Health International
Ifakara Health Institute	

We fed all MDs graduated from 1992 to 2010 into SPSS statistics software by year and institutions of graduation. A frequency distribution table was generated, indicating the number of graduates per year per institution. From the proposal for Health Sector Reforms report released by MoHSW in 1994, Tanzania had 1265 MDs in 1991–1992, before health sector reforms. We used this number as our base line. A review of the 2008–2013 HRH strategic plan identified an attrition rate of less than 3% [15].

$$ {T}_{\mathrm{A}} = \left[\left({T}_{\mathrm{t}} + B\right)\times \left(100- A\right)\%\right] $$

where

*T*_A_ = total number of MDs trained by 2010 (before and after 1992)

*T*_t_ = total number of MDs trained from 1992 to 2010

*A* = attrition rate of HRH in the health sector in Tanzania

*B* = baseline number of MDs before health sector reforms (before 1992)

#### Number of medical doctors available

We conducted a cross-sectional survey on human resources for health from August 2011 to September 2012 to document the number of HRH available in the country by July 2011. The aim of the survey was to locate the available HRH in six selected cadres in the country. The six cadres were specialist doctors (clinical and public health), medical doctors (MDs), pharmacists (B. Pharm), dental surgeons (DDS), assistant medical officers (AMOs) and assistant dental officers (ADOs). The survey collected information on who was at that particular district/institution/organization, when he or she has joined that place, where he or she was before coming to that place, place of graduation and year of graduation. The information was collected by contacting regional medical officers, district medical officers, regional health secretaries, district health secretaries, heads of NGOS, and heads of institutions, agencies and associations (Tables 1 and 2). We gathered information through phone calls, emails and physical visits. The e-mails contained a brief description of the purpose of the survey, the template for completing the information and a letter of permission to conduct the survey from MoHSW.

We analysed survey data with the aid of SPSS statistical software. We then generated frequency tables by categories of districts, regions, consultant hospitals, NGOs, private health facilities and training institutions. The tables were then combined into one frequency table summarizing all the MDs available in Tanzania. In this study MD was defined as any person who had a minimum of degree of Doctor of Medicine (MD, MbChb, MBBS and any other meant to practice medicine) and up to specialization level be it clinical, basic science or public health. We grouped the number of MDs available into zones based on the seven geographical zones in Tanzania.

From the population and housing census, we computed the total population of each zone. From the number of MDs and population available in each zone, we calculated the number of MDs per 1000 population as follows:

$$ \mathrm{M}\mathrm{D}\mathrm{s}:\ 1000\ \mathrm{population} = \frac{\mathrm{Total}\ \mathrm{number}\ \mathrm{of}\ \mathrm{MDs}}{\mathrm{Total}\ \mathrm{population}}\times 1000 $$

We tabulated the number of MDs in each zone, percentage of MDs available in each zone, MDs per 1000 people and the total population in each zone.

#### Recommended, trained and available gap

To document the differences between the actual number of recommended and trained MDs, we subtracted the minimum number of MDs recommended from the cumulative number of MDs trained. We then documented the gap between the number of MDs available and the number trained by subtracting the number available from the cumulative number trained. We plotted the cumulative number of MDs trained in the country after the health sector reforms to include our baseline MDs with the total number of MDs available in different years and with the actual number of MDs recommended in Tanzania in different years.

## Results

### Number of medical doctors recommended

By 2011, Tanzania had 464 health centres, 145 district/designated district/district-level hospitals, 34 regional hospitals or regional-equivalent hospitals, nine referral hospitals and one national hospital. The MoHSW had several units that also require MDs. The number of MDs required to work in all health facilities available in Tanzania by 2011 is summarized in Table 3 below. We did not obtain the number of MDs recommended to work as programme officers in NGOs, which do not own health facilities, and for those to work as university faculty. Table 3 summarizes the number of MDs recommended in Tanzania.

**Table 3:** Number of MDs recommended in staffing levels

Type/Level of facility	Number of health facilities	Number of MDs recommended per facility	Total number of MDs recommended
Health centres (level 1)	464	1	464
District hospitals/designated hospitals (level 2)	145	8–23	1160–3335
Regional hospitals and other RRH (level 3)	34	29–30	986–1020
Referral/specialized hospitals/national hospital (level 4)	10	Varies depending on catchment	607
MoHSW agencies	N/A	Varies depending on responsibilities	81
Allied health training institutions (that require MDs)	8	Varies depending on size of institution	28
Total			3326–5535

Source: United Republic of Tanzania, MoHSW, staffing levels for MoHSW departments, health service facilities, health training institutions and agencies 2014–2018

The total number of MDs recommended to work in Tanzania’s health sector ranges from a minimum of 3326 up to 5535 (Table 3). This is equivalent to a ratio of 0.08–0.13 doctors per 1000 population. This ratio is calculated basing on a total population of 43,625,354 [40]. The minimum threshold recommended by the staffing level in Tanzania corresponds to the minimum threshold recommended by WHO (0.1 doctors per 1000 people) [46].

### Training of medical doctors in Tanzania 1992–2010

From 1992 to 2000, only one institution had a training degree programme for doctors of medicine in Tanzania—the Muhimbili University College of Health Sciences (currently the Muhimbili University of Health and Allied Sciences). The public sector owned this institution. In total, 283 MDs graduated from 1992 to 2000, during which time 91 MDs graduated from schools abroad and registered with the Medical Council of Tanganyika. Therefore, 374 MDs graduated from 1992 to 2000.

From 2001 to 2010, 2248 MDs graduated both locally and abroad (Table 4). Of the 2022 locally trained MDs, about 64% were trained by the public university.

**Table 4:** Medical doctors graduated from 2001 to 2010 in Tanzania

Name of institution	Number of MD graduates per year										Total
	2001	2002	2003	2004	2005	2006	2007	2008	2009	2010	
MUHAS	56	61	105	103	122	134	175	201	173	155	1 285
IMTU	–	15	11	11	11	27	24	24	39	71	233
KCMU	04	12	–	–	39	34	27	–	26	76	218
HKMU	–	–	04	12	20	08	26	42	50	70	232
CUHAS	–	–	–	–	–	–	–	09	24	21	54
Abroad	38	45	16	14	18	15	13	34	18	15	226
Total	60	88	120	126	192	203	252	276	312	393	2 248

Source: graduation books from training institutions and records from the Medical Council of Tanganyika

From 1992 to 2010, 2622 MDs graduated in local universities and abroad, with 67% of the locally trained MDs graduating from the public university. Including our baseline number of 1265 MDs, by 2010 there were 3887 MDs trained in Tanzania and abroad. Taking into account a 3% attrition rate, the total number of MDs supposed to be available in Tanzania by 2011 was 3770. The increase from training is more than double the number of MDs available in the country before the HSRs.

### Deployment of medical doctors in Tanzania

In total, 1299 MDs were working in district, regional and consultant hospitals (both public and private) and in selected NGOs. Our survey covered largely the public sector, FBOs and large NGOs (Table 5).

**Table 5:** Number of MDs available in Tanzania as of July 2011

Place/institution type	Number of facilities	Number of MDs available
Districts	117	309
Regional hospitals	24	123
Referral and consultant hospitals	09	340
Training institutions	15	61
MoHSW units	45	170
NGOs	10	72
FBOs	From 24 regions	224
Total		1 299

Source: field survey

### Geographical distribution of medical doctors

The distribution of MDs in Tanzania is not even; neither the zones nor the national average match the WHO recommended threshold of 0.1 doctors per 1000 people (Table 6). The eastern and northern zones, which have about 30% of the population, host about 50% of the total MDs available in this country. Within regions, we found a wide variation of the MDs available, with a large number concentrating in urban districts. Nineteen of the 117 districts in 12 out of the 20 regions surveyed had no single MD serving the public sector. Within the zones, majority of the MDs were in big cities and district towns. In the eastern zone, 85% of the MDs were in Dar es Salaam. In the southern highlands, 60% of all MDs were in Mbeya, and in Lake zone 62% of all MDs were in Mwanza.

**Table 6:** Distribution of medical doctors per zone and population

Zone	Number of MDs vs population, *N* [%, MD: 1000 population]	Population [2012 Census] *N* [%]
Central zone	56 (4.31, 0.02)	3 454 225 (7.92)
Eastern zone	575 (44.26, 0.07)	7 681 701 (17.61)
Lake zone	159 (12.24, 0.01)	11 832 857 (27.12)
Northern zone	257 (19.78, 0.04)	6 804 733 (15.60)
Southern zone	37 (2.85, 0.02)	2 135 506 (4.90)
Southern highlands	172 (13.24, 0.03)	5 727 636 (13.13)
Western zone	43 (3.31, 0.01)	5 988 696 (13.73)
Total	1 299 (100, 0.03)	43 625 354

Source: field survey

### Trends of the recommended, graduated and available number of medical doctors

**Fig. 2:**
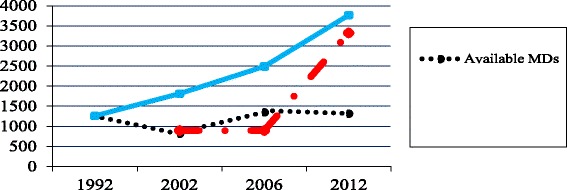
Trends of medical doctors in Tanzania

The number of MDs available by 2012 was about 34% (1299/3770) of all MD graduates in the country by 2010 (Fig. 2). From 1999 to 2012, recommended staffing levels were not revised, making the recommended number of MDs to stand at 897 in all those years. There were 822 MDs in 2002, 1346 MDs in 2006 and 1315 MDs in 2012 working in Tanzania (country profile reports). The number of recommended MDs changed to a minimum of 3326 and up to 5535 after revision of the staffing levels in 2012. Our baseline number is 1265 MDs documented in the proposal for Health Sector Reforms report released by MoHSW in 1994. From 1992 to 2010, 2622 MDs graduated both internally and abroad. Considering the baseline number before health sector reforms and the 3% attrition rate, the number of MDs trained by 2010 and who were supposed to be available in Tanzania is 3770.

## Discussion

The question of critical shortage and maldistribution of HRH is not unique to Tanzania rather it is a global issue. World Health Organization recommends consideration of HRH in three critical stages: entry (training and placement), availability (deployment and motivation) and exit (retention and succession plan) [42]. Among the challenges facing health systems in low- and middle-income countries is the weak HRH planning capacity [47]. Among the challenges which face the HRH planning in Tanzania for many years has been the poor coordination among trainers and employers [15]. This partly contributes to the difficulties in projecting the actual number of HRH to train and deploy. Failure to deploy and retain the trained MDs fuels the brain drain and brain waste [18].

In this study, we aimed to assess the achievements in the training and deployment of medical doctors in Tanzania for a period of about 20 years after the HSRs of the 1990s. The documented number of available MDs from our survey and from other official documents of the Ministry of Health and Social Welfare (MoHSW) is far less compared to the number of MDs trained. From this study, we found that the number of MDs available was 34% of all MD graduates in the country. The findings from the survey are congruent to the number of MDs documented by MoHSW as of 2012 [37, 48].

### Training of medical doctors post the 1990s health sector reforms

After the HSRs, the training of MDs received considerable attention in Tanzania. More than 2000 MDs have been trained in 20 years compared to the number trained before the HSRs. However, the contribution of the private sector in training of MDs has been growing slowly; hence, the key producer has remained the public sector.

During the 20-year period, the private sector contributed about 33% of all MD graduates, four institutions compared to one in the public sector made this contribution. Although medical students have to pay tuition fees in private institutions, the majority are supported through grants from the government [4, 18]. The small number of MDs graduated challenges the support provided by the government to private institutions versus expansion of the public institution. With government support, it was expected that the contribution of the private sector to the training of MDs would have been higher. The documented main reasons for private institutions’ small contribution to number of graduates include low admission capacity due to a lack of space, low capacity teaching hospitals and an overwhelming shortage of staff among the institutions [4].

### Availability of medical doctors

It is undebatable that there has been improvement in the training of MDs in the country; however, their deployment has not yet shown considerable improvements. The process of deployment of MDs in the public sector involves a long chain of stakeholders, which make the process bureaucratic, expensive and time consuming [33]. The MoHSW has no full control over the recruitment of MDs, it is involved in the recruitment process after the vacancies have been approved by other authorities [43]. This does not only make some of the MDs to avoid the public sector but also make it difficult to ensure comprehensive HRH planning. The number of MDs to be recruited in Tanzania is not determined by the need assessment done by the districts; it is determined by the number of vacancies approved by the President Office Public Service Management after approval of funding to those vacancies by the Ministry of Finance and Economic Affairs [34]. Most of the time the number of approved vacancies has been far less compared to the actual need and the number of MDs graduated [18]. Sirili [18] documented that the number of approved vacancies for recruiting MDs from 2006 to 2010 was less by 40% of the number of MDs who finished their internship in that period. The evaluation report by MoHSW in 2014 [34] pointed out that the number of MDs posted to the districts and regions by the MoHSW most of the time is far less than what is requested and sometimes other cadres are posted in places of MDs.

The number of MDs graduating has been increasing steadily with a steep increase from 2006 to 2010. The number of MDs available decreased steadily from 1992 to 2002 (Fig. 1). This might partly be explained by the retrenchment policy that accompanied the freezing of public sector employment due to the budget ceiling from 1993 to 2001 [15, 49]. Revision of the staffing levels after 2010 increased the number of recommended MDs to more than three times that of 1999. The observed trend means that the number of MDs trained would be sufficient to meet the number of recommended MDs in Tanzania at any point in time since 1992 if they were all absorbed in the health sector.

Other studies conducted in Tanzania have captured with high precision the number of HRH in the public sector and FBOs with less precision in the for-profit private facilities [33]. This is also true for our survey which mainly used the regional and district medical officers to capture information on the number of MDs available in their districts.

### Geographical distribution of the available medical doctors

From our survey, majority of the MDs were in big cities and town parts of the districts. Out of the 20 regions surveyed, five regions that host less than 25% of the country’s population contained above 50% of all MDs in the country. The distribution of the available MDs in Tanzania depicts the first form of brain drain—brain drain within the country.

Our findings are in line with what was documented by other studies conducted in Tanzania from 2006 to 2012 [7, 15–17]. Many factors contribute to the rural-urban dynamic including underequipped facilities in rural districts, poor working conditions, lack of housing, interference from political leaders, poor social services, need for good schools for children and lack of places for earning extra income [17, 50]. One of the aims of the health sector reforms was to ensure that the health system became responsive by putting the skilled workforce closer to the people [51]. Our findings send a message that resolving the maldistribution of HRH in Tanzania calls for joint efforts from all development partners.

International brain drain is not a common terminology in Tanzania; it is believed that many MDs work in Tanzania and those who are not absorbed by the public sector are in the private sector. It is assumed that most medical students received government grants and signed a bond agreement to work and stay in Tanzania for 5 years after graduation [52]. Contrary to this assumption, however, an international brain drain of MDs is probably among major contributors of the shortage of MDs in Tanzania [13]. In 2012, Sikika reported that 26% of MDs who were born in Tanzania were working abroad [53].

### Methodological consideration

The staffing level did not document the number recommended to work as university faculty and those required to work as programme officers in the NGOs, which do not own health facilities. Therefore, the minimum number of MDs recommended might be higher than the documented. Our survey data are limited also in concluding that this is the actual number of MDs available in the country. This is because we used the public sector in regions and districts to capture information on available MDs in both the public and private sectors. Our survey was also limited to large NGOs and institutions in the country excluding smaller ones.

However, from the information of MDs available in big cities showed that the majority of the for-profit private hospitals used many part-timers who were employees in public hospitals. Therefore, the number of MDs available in private hospitals that is not captured by our survey is far less compared to the number of MDs yet to be traced. Studies conducted in Tanzania on tracing graduates in private employment often faced many challenges due to regular change of employers [49, 53, 54].

The strengths of our study is based on the use of a conceptual model considering both the demand and supply to guide our study, triangulation of data sources in comparing the number of MDs available by 2011, estimates of the number of MDs per 1000 people using census data gathered just 1 year after our study and analysis of the gap of the number of MDs recommended against those who were trained and available at different points in time using the staffing levels, graduation books, records and country profile reports.

## Conclusions

It is undebatable that there has been a marked increase in number of MDs trained in Tanzania since the 1990s HSRs. These findings are consistent with what other studies have pointed out about the HRH situation in Tanzania. Increasing the number of MDs graduating addresses only one aspect in ensuring their availability.

Comprehensive HRH planning that considers all three stages of entry, availability and exit is needed. Therefore, HRH planning in Tanzania also should consider the capacity to deploy and retain the MDs and other skilled HRH. This will reduce the brain drain and brain waste due to a failure to deploy and retain the skilled HRH. Although our study did not look at the planning capacity of HRH, the findings give a clue on the difficulty of HRH planning in an era of many players in the health sector. With the pressing shortage of faculty in health training institutions, there is a need for examining the quality of these graduates and their trainers to ensure that the increased number of graduates does not compromise their quality.

From this study, we have been able to trace less than half of all graduated MDs since the health sector reforms. No documentation or other official data in or from the MoHSW has been able to show the whereabouts of the other MD graduates during this period. It is important that a reliable and continuous capacity-built HRH audit be carried throughout all health facilities, institutions, organizations and agencies in the country to have a clear database that can be maintained and updated regularly.

Human resources for health planning should consider training, placement and retention. This calls for coordination between the training institutions, professional registration bodies and the employers. Finally, these results call for a consideration of joint planning among the stakeholders in both the public and private sectors.

## References

[1] WHO (2007). Everybody's business-strengthening health systems to improve health outcomes: WHO's framework for action.

[2] Anyangwe SC, Mtonga C (2007). Inequities in the global health workforce: the greatest impediment to health in sub-Saharan Africa. Int J Environ Res Public Health.

[3] Kinfu Y, Dal Poz MR, Mercer H, Evans DB (2009). The health worker shortage in Africa: are enough physicians and nurses being trained?. Bull World Health Organ.

[4] Sirili N, Angwara K, Simba D (2013). Challenges towards realization of health care sector goals of Tanzania development vision 2025, training and deployment of graduate Human resource for health. East Afr J Public Health.

[5] Abimbola S, Olanipekun T, Igbokwe U, Negin J, Jan S, Martiniuk A, Ihebuzor N, Aina M (2015). How decentralisation influences the retention of primary health care workers in rural Nigeria. Glob Health Action.

[6] O'Brien P, Gostin LO (2011). Health worker shortages and global justice. Health Worker Shortages and Global Justice, Millbank Memorial Fund.

[7] Mæstad O. Human resources for health in Tanzania: challenges, policy options and knowledge gaps. CMI report. Bergen: Chr. Michelsen Institute; 2006

[8] MUHAS. Annual Report 2010-2011. Directorate of Planning and Development. Dar es Salaam: Muhimbili University of Health and Allied Sciences; 2011.

[9] USAID. Health systems strengths and weaknesses. Health systems strengths and weaknesses. Health Systems 2020 Tanzania country brief. North Bethesda, Maryland: United States Agency for International Development; 2007.

[10] SIKIKA. Tanzania Health Sector Budget Analysis 2005/06-2011/2012. Dar es Salaam: SIKIKA; 2012.

[11] WHO (2010). WHO Global Code of Practice on the International Recruitment of Health Personnel.

[12] SHOPS Project (2013). Tanzania Private Health Sector Assessment. Brief.

[13] Clemens MA, Pettersson G (2008). New data on African health professionals abroad. Hum Resour Health.

[14] URT. Mid-term analytical review of the performance of the health sector strategic plan III 2009-2015. Dar es Salaam: United Republic of Tanzania; 2013.

[15] URT. Ministry of Health and Social Welfare. Human Resource for Health Strategic Plan 2008-2013. Dar es Salaam: United Republic of Tanzania; 2007

[16] Munga MA, Mæstad O (2009). Measuring inequalities in the distribution of health workers: the case of Tanzania. Hum Resour Health.

[17] Manzi F, Schellenberg JA, Hutton G, Wyss K, Mbuya C, Shirima K, Mshinda H, Tanner M, Schellenberg D (2012). Human resources for health care delivery in Tanzania: a multifaceted problem. Hum Resour Health.

[18] Sirili N, Kiwara A, Nyongole O, Frumence G, Semakafu A, Hurtig AK (2014). Addressing the human resource for health crisis in Tanzania: the lost in transition syndrome. Tanzan J Health Res.

[19] Ishijima H, Mapunda M, Mndeme M, Sukums F, Mlay VS (2015). Challenges and opportunities for effective adoption of HRH information systems in developing countries: national rollout of HRHIS and TIIS in Tanzania. Hum Resour Health.

[20] Tanzania MF (2007). Joint External Evaluation of the Health Sector in Tanzania: 1999-2006.

[21] Berman P, Bossert T (2000). A decade of health sector reform in developing countries: what have we learned.

[22] Therkildsen O (2000). Public sector reform in a poor, aid-dependent country, Tanzania. Public Administration & Development.

[23] Cassels A (1995). Health sector reform: key issues in less developed countries. J Int Dev.

[24] URT. An Act to amend the Private Hospitals (Regulation) Act, 1977, to make provision for the management of private hospitals by individuals and organizations. Dar es Salaam: United Republic of Tanzania; 1992.

[25] Maluka SO, Hurtig AK, Sebastián MS, Shayo E, Byskov J, Kamuzora P (2011). Decentralization and health care prioritization process in Tanzania: from national rhetoric to local reality. Int J Health Plann Manage.

[26] URT. Policy Paper on Local Government reforms. Dar es Salaam: United Republic of Tanzania; 1998.

[27] Itika J, Mashindano O, Kessy FL (2011). Successes and Constraints for Improving Public Private Partnership in Health Services Delivery in Tanzania. Economic and Social Researches Foundation.

[28] URT. The private hospitals (regulation) act, 1977. Dar es Salaam: United Republic of Tanzania; 1977.

[29] Hutchinson P. Decentralization in Tanzania: the view of District Health Management Teams. Chapel Hill: Carolina Population Center University of North Carolina at Chapel Hill; 2002.

[30] Mkony CA (2012). Emergence of a university of health sciences: health professions education in Tanzania. J Public Health Policy.

[31] MUHAS. Triennium report 2006/07-2008/09. Directorate of Planning and Development. Dar es Salaam: Muhimbili University of Health and Allied Sciences; 2009.

[32] Munga MA, Songstad NG, Blystad A, Mæstad O (2009). The decentralisation-centralisation dilemma: recruitment and distribution of health workers in remote districts of Tanzania. BMC International Health and Human Rights.

[33] URT. Ministry of Health and Social Welfare. Mid Term Review of the Health Sector Strategic Plan III 2009-2015 Main report. Dar es Salaam: United Republic of Tanzania; 2015.

[34] URT. Ministry of Health and Social Welfare. Human Resource for Health and Social welfare Country Profile 2013/2014. Dar es Salaam: United Republic of Tanzania; 2014.

[35] Balati J. Christian Social Services Commission. ACHAP meeting Strengthening PPPs and Inter-faith partnerships for Universal Health Coverage. Dar es Salaam: Christian Social Services Commission; 2015.

[36] KCMC. Annual Report 2014. Kilimanjaro Christian Medical College. 2014

[37] URT. Ministry of Health and Social Welfare. Human Resource for Health and Social welfare Country Profile 2012/2013. Dar es Salaam: United Republic of Tanzania; 2013.

[38] Juma A, Kangalawe GA, Dalrymple E, Kanyenda T. Case Study #9-11, "Brain Drain of Health Professionals in Tanzania". In: Pinstrup-Andersen P, Cheng F, editors. "Food Policy for Developing Countries: Case Studies." 19 pp. 2012. URL., http://cip.cornell.edu/dns.gfs/1351876999.

[39] URT. Ministry of Health and Social Welfare. Nationa health policy in Tanzania. Dar es Salaam: United Republic of Tanzania; 1990.

[40] URT.National Bureau of Statistics. 2012 population and housing census. Population distribution by administrative areas. Dar es Salaam: United Republic of Tanzania; 2013.

[41] Yin RK (2003). Case Study Research: Design and Methods. Applied Social Research Methods Series.

[42] WHO (2006). The world health report: 2006: working together for health.

[43] URT. Ministry of Health and Social Welfare Final Evaluation Report for Human Resource for Health Strategic Plan 2008-2013. Dar es Salaam: United Republic of Tanzania; 2014.

[44] O’Brien-Pallas L, Baumann A, Donner G, Murphy GT, Lochhaas-Gerlach J, Luba M (2001). Forecasting models for human resources in health care. J Adv Nurs.

[45] URT. Ministry of Health and Social Welfare. Staffing Levels for Ministry of Health and Social Welfare Departments, Health Service Facilities, Health Training Institutions and Agencies 2014-2018. Dar es Salaam: United Republic of Tanzania; 2013.

[46] WHO. World Health Statistics 2010. Geneva: World Health Organization. 2010;177.

[47] Kolehmainen-Aitken RL. Human resources planning: issues and methods. Boston, Massachusetts: Data for Decision Making Project, Department of Population and International Health, Harvard School of Public Health; 1993.

[48] Bott R (2014). Number of health workers by regions, 2013. Igarss.

[49] Kwesigabo G, Mwangu MA, Kakoko DC, Warriner I, Mkony CA, Killewo J, Macfarlane SB, Kaaya EE, Freeman P (2012). Tanzania's health system and workforce crisis. J Public Health Policy.

[50] Yumkella F (2005). Retention: health workforce issues and response actions in low-resource settings. Capacity project resource paper.

[51] URT. Ministry of Health and Social Welfare. National Health Policy [Draft]. Dar es Salaam: United Republic of Tanzania; 2003.

[52] URT (2012). Ministry of education and vocation training. Higher education students loans board. Local undergraduate application form for grants.

[53] Sikika and MAT. Where are the Doctors ? - Tracking Study of Medical Doctors. Dar es Salaam: SIKIKA; 2013.

[54] Pemba S, Macfarlane SB, Mpembeni R, Goodell AJ, Kaaya EE (2012). Tracking university graduates in the workforce: information to improve education and health systems in Tanzania. J Public Health Policy.

